# A physical fitness profile of state highway patrol officers by gender and age

**DOI:** 10.1186/s40557-017-0173-0

**Published:** 2017-06-01

**Authors:** J. Jay Dawes, Robin M. Orr, Richard R. Flores, Robert G. Lockie, Charlie Kornhauser, Ryan Holmes

**Affiliations:** 1 Department of Health Sciences, University of Colorado Colorado Springs, 1420 Austin Bluffs Pkwy, Colorado Springs, CO 80918 USA; 2 Tactical Research Unit, Bond University, Robina, Gold Coast, 4226 QLD Australia; 3 Department of Kinesiology, Cal State Fullerton University, 800 N. State College Blvd., Fullerton, CA 92834 USA; 4 Colorado State Highway Patrol, Training Academy, 700 Kipling Street, Lakewood, CO 80215 USA

**Keywords:** Police, Law enforcement, Occupational fitness

## Abstract

**Background:**

Law enforcement officers perform physically demanding tasks that generally remain constant as they age. However, there is limited population-specific research on age, gender and normative fitness values for law enforcement officers as opposed to those of the general population. The purpose of this study was to profile the current level of fitness for highway patrol officers based on age and gender and provide percentile ranking charts unique to this population.

**Methods:**

Retrospective data for six-hundred and thirty-one state troopers (♂ = 597; mean age = 39.52 ± 8.09 yrs; mean height = 180.72 ± 7.06 cm; mean weight = 93.66 ± 15.72 kg: ♀ = 34; mean age = 36.20 ± 8.45 years; mean height = 169.62 ± 6.65 cm; mean weight = 74.02 ± 14.91 kg) collected in 2014–2015 were provided for analysis. Data included demographic (age), anthropometric (height and weight), and select fitness (VJ, push-ups, sit ups, isometric leg/back strength, isometric grip strength and 20 m shuttle run test) information.

**Results:**

There were generally significant differences between genders for all anthropometric and fitness measures, most consistently in the 30–39 age groups. While there was a general decline in push-up and shuttle run performance in female officers, these results did not reach significance. For male officers, there were significant differences between the 20–29 year-old age group and the 30–39, 40–49 and 50–59 year-old groups with the younger group performing better in VJ, push-ups, sit ups and number of shuttle runs than the older groups. There were no differences in isometric grip strength and leg back dynamometer measures between age groups.

**Conclusion:**

Male officers tended to be heavier, taller and perform significantly better than female officers in all measures bar sit-ups. While there appeared to be a general decline in certain physical characteristics across genders with increasing age the notable differences were between the youngest male age group (20–29 years) and all other male age groups with a potential reason being the lack of fitness requirements once typically younger cadets leave the academy. Percentile rankings for the assessed measures were found to have elements very specific to this population when compared to the general population and those provided in this paper can be used to inform future profiling and research in this population.

## Background

Law enforcement officers (LEOs) can be called upon, at a moment’s notice, at any hour of the day to serve the citizens they have sworn to protect [1]. Provision of this service can see LEOs perform physically and mentally demanding tasks in an occupation that is often spent in a sedentary position punctuated with bursts of physical exertion [1, 2]. These demanding tasks may include, but are not limited to, pursuing and apprehending a suspect, forcing entry during a search warrant, close encounter hand-to-hand combat, lifting heavy objects, and maneuvering quickly on foot to a situation [1]. As such, performance of these tasks require the officer to possess an adequate base of cardiorespiratory and muscular fitness in order to perform these duties in an effective manner [3]. In addition to these abrupt physical exertions, LEOs can spend a good portion of their work shifts sitting in a patrol car or at a desk completing reports [1]. The combination of this sedentary behavior, and potential for the associated negative impact on physical fitness, coupled with near maximal exertions may increase ones risk for injury, morbidity, and mortality. It is therefore not surprising that LEOs have been found to be twice as likely to suffer from cardiovascular disease as the general population [4]. Therefore, the need to attain and maintain physical fitness is imperative to an officer’s overall health and ability to perform job-tasks safely and effectively throughout their career [1]. Furthermore, it is suggested that LEOs need to be more physically fit than the general population [5].

Spitler et al. [3] measured and evaluated oxygen uptake, body composition, and muscular strength, endurance and power for 12 police officers. The researchers discovered that the officers displayed average to above average rankings in health and physical fitness when compared to age and gender based norms in their respective age groups [3]. While the small sample size in this study limits inferences to the larger law enforcement population, this work is supported by other research [6, 7]. For example, a study by Dawes et al. [7] observed that LEOs generally maintained their push-up ability as they aged, notably increasing their capability above that of the general population age comparative norms. In this study increases in percentage of body fat, which increased with age, was the most strongly negatively correlated (*r* = -.540, *p* < .001) factor for push-up performance decline as opposed to age alone (*p* = .330). There is however conflicting research. Sorensen et al. [1] found that Finnish LEOs decreased significantly decreased in push-up ability over a 15-year period (mean difference -3.4, *p* < .001) as well as VO2max, pull up and sit up performance. Furthermore, Sorensen et al. [1] suggested that high levels of stress and a lack of physical fitness in LEOs may lead to a decline in health and fitness over the course of their career.

Acknowledging these conflicting findings, it is imperative to appreciate that as an officer ages the physical demands of patrolling and performing essential job tasks generally remain constant. As such, regardless of age, maintenance of fitness is critical to ensure safety for the officer, their teammates, and the public [8, 9]. On this basis, minimal research has examined the physical and physiological characteristics of police officers with a large enough sample size to establish normative, or reference, data within this population.

Establishing population-specific normative values will allow for the comparison of LEO officers to the general population and may also provide greater insight into population-related differences in fitness. Furthermore, through establishing normative values, greater insight for developing strength and conditioning programs to improve, or maintain, fitness over the course of an officer’s career can be gained. Therefore, the purpose of this investigation was to profile the current level of fitness for highway patrol officers based on age and gender and provide percentile ranking charts unique to this population.

## Methods

Retrospective data for six-hundred and thirty-one state troopers (♂ = 597; mean age = 39.52 ± 8.09 years; mean height = 180.72 ± 7.06 cm; mean weight = 93.66 ± 15.72 kg: ♀ = 34; mean age = 36.20 ± 8.45 years; mean height = 169.62 ± 6.65 cm; mean weight = 74.02 ± 14.91 kg) were provided for analysis. This data were collected in 2014–2015 as part of the agencies normal yearly fitness assessment. Prior to the analysis, approval for the use of the data was obtained from the University of Colorado Colorado Springs Institutional Review Board (IRB 15-074) for human subjects and the Bond University Human Research Ethics Committee (RO 1927).

This data included demographic (age) and anthropometric (height and weight) information as well as select fitness measures (VJ, push-ups, sit ups, isometric leg/back strength, isometric grip strength and 20 m shuttle run test), and was collected by the state patrol training staff.

### Age, height and weight

Age (yrs), height (in) and weight (lbs) measurements for incumbents were self-reported by the state troopers. All imperial measures were subsequently converted to metric values for analysis.

### Vertical jump

Vertical jump (VJ) height provides an indirect measure of lower-body power [10], and thus features in many testing batteries for LEOs [2, 11–15]. VJ height was measured using a Just Jump (ProBotics Inc, Huntsville, Al) electrical contact operated system. The Just Jump Mat is a 27-in. x 27-in. mat that calculates VJ height by measuring vertical displacement time. Vertical jump height for this device was calculated by measuring the amount of time the feet are not in contact with the mat. All troopers were instructed to step on the mat, and when ready perform a countermovement arm swing and jump as high as possible. This score was used to determine the VJ height of each trooper. The best of 3 attempts were taken and maximal jump height was recorded to the nearest 0.5 in. before being converted into metric units.

### 1-Minute push-ups

The 1-min push-up test is commonly completed by law enforcement officers and provides a measure of endurance for the upper-body muscles [11–14, 16]. This test also provides an indication of relative strength and the ability to move the body weight [17, 18]. In this test, all troopers had been required to begin the test in the standard “up” position with the body rigid and straight, the hands positioned slightly wider than shoulder-width apart and the fingers pointed forward. A partner then placed a fist on the floor directly under the individual’s chest. On the “go” command, the tester began the stopwatch and the participant would bend their elbows, lowering themselves until their chest was in contact with their partner’s fist and then extend the elbows until back in the “up” position. The troopers then proceeded to perform as many push-ups as possible in the time allotted using this technique. Troopers were allowed to rest in the straight-arm position, as long a neutral trunk position was maintained. The test was terminated when a trooper was unable to perform this movement with proper technique, or when the one-minute time limit expired.

### 1-Minute sit-ups

All troopers were required to begin the assessment lying in a supine position, with the knees bent, feet flat on the ground and the arms positioned in front of the body with arms wrapped across the chest and each hand on the opposite shoulder. Once in position the participant will flex the trunk, elevating the shoulders off the floor until the elbows touch the knees. During this assessment each trooper had a partner anchor their feet in place to assist in keeping the feet flat on the floor throughout the exercise movement. On the “go” command, the tester began the stopwatch and the participant began the assessment. The troopers then proceeded to perform as many sit-ups as possible in 1-min using this technique.

### Isometric leg/back strength

Although potentially not as precise an isometric mid-thigh pull on a force plate, the leg/back chain dynamometer does provide the advantage of being less expensive, more efficient, and easily transportable, and still capable of providing quantitative data [19]. This assessment has been used as a measure of strength in athletic populations [20]. Considering this, isometric a leg/back chain dynamometer (Medico Inc., Phoenix, Az.) was used to measure the strength of the legs and lower back. The chain, which connects the scale on one end and a handle on the other, was adjusted so that the trooper’s knees were bent at approximately 110°. While maintaining good spinal posture, straight arms and feet flat on the base of the dynamometer, the troopers pulled the handle upward as hard as possible by extending through the hips and knees. This dynamometer was calibrated within .05 kg using an industrial portable digital hanging scale prior to use. Troopers were allowed a single trial and their score to the nearest pound was recorded.

### Grip strength

Dominant hand grip strength was measured using a handgrip dynamometer (Takei Scientific Instruments, Japan). The dynamometer was adjusted so that the base of the first metacarpal and the middle four fingers were in contact with the handle. Troopers were then instructed to squeeze the handle as hard as possible. One attempt was allowed and the sore was recorded to the nearest kilogram.

### 20 Meter Multi-Stage Fitness Test (20 m-MSFT)

Troopers were required to run back and forth between two lines marked on the ground spaced exactly 20 meters apart. The speed of running for this test is standardized by pre-recorded auditory cues (beeps). The initial speed for the test is set at 8.5 km/h and increases by 0.5 km/h with each additional stage. This test is scored according to the final stage and shuttle (e.g. Stage 5.5) the participant is able to achieve before being unable to run at the speed required. The test was terminated when the participant was unable to reach the next line twice in a row in accordance with the auditory cues. Final scores by stage and shuttle were converted for total number of shuttles completed.

### Data management and statistical analysis

The retrospective data were divided into five separate age groups in order to compare trooper fitness scores on push-ups, sit-ups, grip strength and aerobic fitness to age and gender norms in the general population. These groups consisted of 1: 20–29 years. (*n* = 89); Group 2: 30–39 years. (*n* = 218); Group 3: 40–49 years. (*n* = 226); Group 4: 50–59 years. (*n* = 57); and Group 5: 60–69 (*n* = 5). The data were analyzed both by gender, to identify any gender specific differences, but also as pooled data to investigate absolute age based requirements regardless of gender. Using the SPSS 23.0 software package, a descriptive statistical analysis was conducted to determine mean fitness scores for the entire sample with independent samples t-tests used to compare results by gender and by gender within each age grouping. Additionally, a one-way analysis of variance (ANOVA), with pairwise comparisons was used to compare mean differences in fitness scores between age categories within gender. If a significant difference between groups was found within the ANOVA, a Bonferroni post hoc adjustment was used to determine where the significance lay. The level of significance was set at *p* ≤ 0.01 to control for family-wise error. In addition, SPSS was used to create percentile charts for future comparison. Due to the small size of Group 5, the data from this group were excluded from statistical analysis and provided for future reference only.

## Results

Not all officers performed every fitness test due to individual injury status and willingness to participate. Descriptive data and all fitness tests for the entire sample and by gender are displayed in Table 1. As there were only 5 participants in Group 5 (male only) their data were removed for inferential analysis. While there were no significant differences between gender groups in age (*p* = 0.021) or sit up repetitions (*p* = 0.064), there were significant differences across all other measures (Table 1). When considered by age groups there were significant differences between genders with some exceptions. In the oldest group, 50–59 year olds, there were no significant differences between genders while in the 20–29 year and 40–49-year age groups there were no significant differences in sit up performance (*p* = 0.425 and *p* = 0.775 respectively), and shuttle run performance (*p* = 0.11 and *p* = 0.113 respectively). In addition, no significant differences between genders were noted in VO2 between the 40–49-year-old age group (*p* = 0.79). Of note, the only age group where there were significant differences between genders for all measures were the 30–39-year-old age group) (See Table 2).

**Table 1:** Descriptive data and fitness test results by gender

Measure	Female officers	Male officers
Age (yrs) ♀ = 34 ♂ = 597	36.21 ± 8.45	39.52 ± 8.09
Weight (kg) ♀ = 31 ♂ = 587	67.49 ± 25.62	91.99 ± 19.54^a^
Height (cm) ♀ = 33 ♂ = 588	164.65 ± 29.82	177.98 ± 23.13^a^
Vertical Jump (cm.) ♀ = 33 ♂ = 588	36.80 ± 5.69	50.74 ± 8.89^a^
Leg/Back Dynomometer (kgk ♀ = 33 ♂ = 592	116.53 ± 20.85	170.68 ± 37.46^a^
Grip (Kg) ♀ = 32 ♂ = 589	37.875 ± 5.34	55.04 ± 7.77^a^
Push-ups (repetitions) ♀ = 29 ♂ = 582	24.24 ± 11.63	39.09 ± 15.61^a^
Sit-ups (repetitions) ♀ = 33 ♂ = 583	31.06 ± 9.52	34.46 ± 10.29
Shuttles (number) ♀ = 31 ♂ = 550	26.19 ± 10.86	38.04 ± 19.87^a^

^a^Significantly different from female officers at ≤ .001

**Table 2:** Descriptive data and fitness test results by gender stratified by age

Age	Measure	Group population	Female officers	Male officers
20–29 Group 1	Weight (kg) *n* = 89: ♀ = 6: ♂ = 83	83.82 ± 16.38	69.55 ± 15.69^*^	84.85 ± 16.03
	Height (cm) *n* = 89: ♀ = 6: ♂ = 83	179.14 ± 7.78	167.64 ± 7.18^*^	179.97 ± 7.17
	Vertical Jump (cm.) *n* = 88: ♀ = 6: ♂ = 82	57.25 ± 9.68	40.46 ± 8.13^*^	58.47 ± 8.79
	Grip (kg) *n* = 87: ♀ = 6: ♂ = 81	53.53 ± 8.49	37.67 ± 5.57^*^	54.67 ± 7.47
	Push-ups (repetitions) *n* = 88: ♀ = 6: ♂ = 82	46.52 ± 15.07	30.50 ± 9.95^*^	47.70 ± 14.74
	Sit-ups (repetitions) *n* = 89: ♀ = 6: ♂ = 83	40.98 ± 8.35	38.33 ± 10.56	41.17 ± 8.22
	Leg/Back Dynomometer (kg) *n* = 89: ♀ = 6: ♂ = 83	169.50 ± 42.27	109.85 ± 26.69^*^	173.81 ± 39.94
	Shuttles (number) *n* = 86: ♀ = 6: ♂ = 80	54.07 ± 21.00	33.33 ± 6.41	55.63 ± 20.90
30–39 Group 2	Weight (kg) *n* = 218: ♀ = 16: ♂ = 202	89.32 ± 19.73	63.50 ± 28.87^*^	91.37 ± 17.35
	Height (cm) *n* = 218: ♀ = 16: ♂ = 202	177.83 ± 22.46	159.23 ± 43.07^*^	179.30 ± 19.40
	Vertical Jump (cm) *n* = 215: ♀ = 16: ♂ = 199	51.49 ± 9.02	36.00 ± 5.82^*^	52.73 ± 8.03^†^
	Grip (kg) *n* = 214: ♀ = 15: ♂ = 199	54.65 ± 9.40	37.20 ± 4.51^*^	55.97 ± 8.30
	Push-ups (repetitions) *n* = 213: ♀ = 15: ♂ = 198	39.44 ± 15.44	25.13 ± 13.05^*^	40.52 ± 14.96^†^
	Sit-ups (repetitions) *n* = 212: ♀ = 16: ♂ = 196	36.04 ± 9.93	28.81 ± 10.51^*^	36.63 ± 9.67^†^
	Leg/Back Dynomometer (kg) *n* = 201: ♀ = 16: ♂ = 200	166.56 ± 38.86	113.35 ± 12.22^*^	170.81 ± 37.08
	Shuttles (number) *n* = 201: ♀ = 15: ♂ = 186	40.98 ± 19.84	25.93 ± 12.57^*^	42.19 ± 19.85^†^
40–49 Group 3	Weight (kg) *n* = 262: ♀ = 10: ♂ = 252	94.34 ± 20.51	69.13 ± 27.64^*^	95.34 ± 19.59^†^
	Height (cm) *n* = 262: ♀ = 10: ♂ = 252	176.02 ± 27.88	170.18 ± 5.35^*^	176.25 ± 28.38
	Vertical Jump (cm. *n* = 258: ♀ = 9: ♂ = 249	47.80 ± 7.70	34.95 ± 5.13^*^	48.29 ± 7.37^†^
	Grip (kg) *n* = 259: ♀ = 9: ♂ = 250	54.46 ± 8.01	36.89 ± 5.06^*^	55.09 ± 7.36
	Push-ups (repetitions) *n* = 252: ♀ = 6: ♂ = 246	36.22 ± 15.53	16.83 ± 3.66^*^	36.70 ± 15.41^†^
	Sit-ups (repetitions) *n* = 256: ♀ = 9: ♂ = 247	31.70 ± 9.82	30.78 ± 5.83	31.73 ± 9.94^†^
	Leg/Back Dynomometer (kg) *n* = 258: ♀ = 9: ♂ = 249	168.94 ± 38.53	118.43 ± 24.28^*^	170.76 ± 37.73
	Shuttles (number) *n* = 237: ♀ = 8: ♂ = 229	31.01 ± 15.43	22.50 ± 10.30	31.31 ± 15.52^†^
50–59 Group 4	Weight (kg) *n* = 57: ♀ = 2: ♂ = 55	89.76 ± 27.35	85.05 ± 11.23	89.94 ± 27.79^†^
	Height (cm) *n* = 57: ♀ = 2: ♂ = 55	178.20 ± 25.06	171.45 ± 8.98	178.45 ± 25.46
	Vertical Jump (cm) *n* = 56: ♀ = 2: ♂ = 54	43.66 ± 8.18	40.51 ± 10.59	43.79 ± 8.18^†^
	Grip (kg) *n* = 54: ♀ = 2: ♂ = 52	52.11 ± 7.68	48.00 ± 4.24	52.27 ± 7.76
	Push-ups (repetitions) *n* = 54: ♀ = 2: ♂ = 52	31.15 ± 14.42	21.00 ± 15.56	31.54 ± 14.39^†^
	Sit-ups (repetitions) *n* = 55: ♀ = 2: ♂ = 53	29.62 ± 9.58	28.50 ± 2.12	29.66 ± 9.76^†^
	Leg/Back Dynomometer (kg) *n* = 57: ♀ = 2: ♂ = 55	164.79 ± 34.84	153.41 ± 14.46	165.21 ± 35.36
	Shuttles (number) *n* = 52: ♀ = 2: ♂ = 50	26.54 ± 13.00	21.50 ± 4.95	26.74 ± 13.20^†^
60–69 Group 5	Weight (kg) *n* = 5: ♀ = 0: ♂ = 5	89.36 ± 11.06	-	89.36 ± 11.06
	Height (cm) *n* = 5: ♀ = 0: ♂ = 5	173.23 ± 6.57	-	173.23 ± 6.57
	Vertical Jump (cm.) *n* = 4: ♀ = 0: ♂ = 4	40.34 ± 4.39	-	40.34 ± 4.39
	Grip (kg) *n* = 5: ♀ = 0: ♂ = 5	50.20 ± 3.27	-	50.20 ± 3.27
	Push-ups (repetitions) *n* = 5: ♀ = 0: ♂ = 5	39.20 ± 12.68	-	39.20 ± 12.68
	Sit-ups (repetitions) *n* = 5: ♀ = 0: ♂ = 5	25.40 ± 11.89	-	25.40 ± 11.89
	Leg/Back Dynomometer (kg) *n* = 5: ♀ = 0: ♂ = 5	169.55 ± 20.62	-	169.55 ± 20.62
	Shuttles (number) *n* = 5: ♀ = 0: ♂ = 5	23.40 ± 7.16	-	23.40 ± 7.16

*Significantly different from male officers at ≤ .01, ^†^Significantly different from 20–29 years old, *p* < .01

When comparing each gender by their own age group (Table 2) there were no significant differences between anthropometric or performance results for any of the female age groups. While some general trends towards a reduction in performance did appear, for example a general reduction in push-up and shuttle run performance, these declines in performance did not reach significance. It should also be noted that female grip strength did approach the stringent level significance required for this study (*p* = 0.042) with the older group (Group 5: 50–59 years, *n* = 38) increasing in strength. However significant differences did exist across all age groups in VJ (*p* < 0.001), push-up (*p* < 0.001), sit up (*p* < 0.001), shuttle run (*p* < 0.001) and VO2 (*p* < 0.001) results for male officers. In addition, while significant differences did exist in weight among male officers (*p* < 0.001) the Bonferroni post hoc assessment identified that the differences in weight existed only between the 20–29-year-old group and both the 40–49-year-old (*p* < 0.001) and 50–59-year-old (*p* = 0.006) groups.

Given the lower number of female participants, especially when stratified across age groups, percentile ranking charts were constructed for only male police officers. The percentile ranking charts VJ (see Table 3), grip strength (see Table 4), push-ups (see Table 5), sit-ups (See Table 6), leg back dynamometer (see Table 7) and number of shuttles (See Table 8) are shown below.

**Table 3:** Percentile ranking of male police officer VJ ability (cm)

Age group (*n*)	20–29 (*n* = 82)	30–39 (*n* = 202)	40–49 (*n* = 247)	50–59 (*n* = 54)
Mean (SD)	58.47 (8.79)	52.73 (8.03)	48.29 (7.37)	43.79 (8.18)
Percentile				
95	72.89	65.90	60.38	57.21
90	69.72	63.01	57.72	54.26
85	67.61	61.08	55.95	52.30
80	65.85	59.48	54.48	50.66
75	64.36	58.11	53.23	49.27
70	63.04	56.91	52.12	48.04
65	61.90	55.86	51.16	46.98
60	60.67	54.74	50.13	45.84
55	59.61	53.77	49.25	44.85
50	58.47	52.73	48.29	43.79
45	57.33	51.69	47.33	42.73
40	56.27	50.72	46.45	41.75
35	55.04	49.60	45.42	40.60
30	53.90	48.55	44.46	39.54
25	52.58	47.35	43.35	38.31
20	51.09	45.98	42.10	36.92
15	49.33	44.38	40.63	35.28
10	47.22	42.45	38.86	33.32
5	44.05	39.56	36.20	30.37

**Table 4:** Percentile ranking of male police officer grip strength ability (kg)

Age group (*n*)	20–29 (*n* = 83)	30–39 (*n* = 199)	40–49 (*n* = 250)	50–59 (*n* = 52)
Mean (SD)	54.67 (7.47)	55.97 (8.30)	55.09 (7.36)	52.27 (7.76)
Percentile				
95	66.92	69.58	67.16	65.00
90	64.23	66.59	64.51	62.20
85	62.44	64.60	62.74	60.34
80	60.94	62.94	61.27	58.79
75	59.67	61.53	60.02	57.47
70	58.55	60.29	58.92	56.31
65	57.58	59.21	57.96	55.30
60	56.54	58.05	56.93	54.21
55	55.64	57.05	56.05	53.28
50	54.67	55.97	55.09	52.27
45	53.70	54.89	54.13	51.26
40	52.80	53.90	53.25	50.33
35	51.76	52.73	52.22	49.24
30	50.79	51.65	51.26	48.23
25	49.67	50.41	50.16	47.07
20	48.40	49.00	48.91	45.75
15	46.90	47.34	47.44	44.20
10	45.11	45.35	45.67	42.34
5	42.42	42.36	43.02	39.54

**Table 5:** Percentile ranking of male police officer push-up ability (repetitions)

Age group (*n*)	20–29 (*n* = 82)	30–39 (*n* = 198)	40–49 (*n* = 246)	50–59 (*n* = 52)
Mean (SD)	47.70 (14.74)	40.52 (14.96)	36.70 (15.41)	31.54 (14.39)
Percentile				
95	71.81	65.05	61.97	55.14
90	66.52	59.67	56.42	49.96
85	62.99	56.08	52.73	46.51
80	60.05	53.09	49.64	43.63
75	57.55	50.54	47.02	41.18
70	55.34	48.30	44.71	39.02
65	53.43	46.35	42.71	37.15
60	51.38	44.26	40.55	35.14
55	49.61	42.46	38.70	33.41
50	47.70	40.52	36.70	31.54
45	45.79	38.58	34.70	29.67
40	44.03	36.78	32.85	27.94
35	41.97	34.69	30.69	25.93
30	40.06	32.74	28.69	24.06
25	37.85	30.50	26.38	21.90
20	35.35	27.95	23.76	19.45
15	32.41	24.96	20.67	16.57
10	28.88	21.37	16.98	13.12
5	23.59	15.99	11.43	7.94

**Table 6:** Percentile ranking of male police officer sit up ability (repetitions)

Age group (*n*)	20–29 (*n* = 83)	30–39 (*n* = 196)	40–49 (*n* = 247)	50–59 (*n* = 53)
Mean (SD)	41.17 (8.22)	36.63 (9.67)	31.73 (9.94)	29.66 (9.76)
Percentile				
95	54.65	52.49	48.03	45.67
90	51.69	49.01	44.45	42.15
85	49.72	46.69	42.07	39.81
80	48.07	44.75	40.08	37.86
75	46.68	43.11	38.39	36.20
70	45.44	41.66	36.90	34.74
65	44.38	40.40	35.61	33.47
60	43.23	39.05	34.22	32.10
55	42.24	37.89	33.02	30.93
50	41.17	36.63	31.73	29.66
45	40.10	35.37	30.44	28.39
40	39.12	34.21	29.25	27.22
35	37.96	32.86	27.85	25.85
30	36.90	31.60	26.56	24.58
25	35.66	30.15	25.07	23.12
20	34.27	28.51	23.38	21.46
15	32.62	26.57	21.39	19.51
10	30.65	24.25	19.01	17.17
5	27.69	20.77	15.43	13.65

**Table 7:** Percentile ranking of male police officer leg back dynamometer ability (kg)

Age group (*n*)	20–29 (*n* = 83)	30–39 (*n* = 200)	40–49 (*n* = 247)	50–59 (*n* = 55)
Mean (SD)	173.81 (39.94)	170.81 (37.08)	170.76 (37.73)	165.21 (35.36)
Percentile				
95	239.31	231.62	232.64	223.20
90	224.93	218.27	219.05	210.47
85	215.35	209.37	210.00	201.98
80	207.36	201.96	202.45	194.91
75	200.57	195.65	196.04	188.90
70	194.58	190.09	190.38	183.60
65	189.39	185.27	185.47	179.00
60	183.80	180.08	180.19	174.05
55	179.00	175.63	175.66	169.81
50	173.81	170.81	170.76	165.21
45	168.62	165.99	165.86	160.61
40	163.83	161.54	161.33	156.37
35	158.23	156.35	156.05	151.42
30	153.04	151.53	151.14	146.82
25	147.05	145.97	145.48	141.52
20	140.26	139.66	139.07	135.51
15	132.27	132.25	131.52	128.44
10	122.69	123.35	122.47	119.95
5	108.31	110.00	108.88	107.22

**Table 8:** Percentile ranking of male police shuttle run ability (number of shuttles)

Age group (*n*)	20–29 (*n* = 80)	30–39 (*n* = 194)	40–49 (*n* = 229)	50–59 (*n* = 50)
Mean (SD)	55.63 (20.90)	42.19 (19.85)	31.31 (15.52)	26.74 (13.20)
Percentile				
95	89.91	74.74	56.76	48.39
90	82.38	67.60	51.18	43.64
85	77.37	62.83	47.45	40.47
80	73.19	58.86	44.35	37.83
75	69.63	55.49	41.71	35.58
70	66.50	52.51	39.38	33.60
65	63.78	49.93	37.36	31.89
60	60.86	47.15	35.19	30.04
55	58.35	44.77	33.33	28.46
50	55.63	42.19	31.31	26.74
45	52.91	39.61	29.29	25.02
40	50.41	37.23	27.43	23.44
35	47.48	34.45	25.26	21.59
30	44.76	31.87	23.24	19.88
25	41.63	28.89	20.91	17.90
20	38.07	25.52	18.27	15.65
15	33.89	21.55	15.17	13.01
10	28.88	16.78	11.44	9.84
5	21.35	9.64	5.86	5.09

## Discussion

The purpose of this investigation was to profile the current level of fitness for highway patrol officers based on age and gender and provide percentile ranking charts unique to this population. With regards to the between-gender comparisons, it was discovered that, on average, male officers, were heavier, taller and displayed greater lower limb power, dominant hand grip strength, upper limb muscular endurance and metabolic fitness than female officers. No significant differences were found between genders in trunk muscular endurance except between the 30–39-year age groups. For the age analysis, there was a general decline in mean performance between male officer age groups in weight, VJ, push-ups, sit ups and number of shuttles completed. However, these differences were generally only significant between those between 20–29 years of age and age groups ranging from 30 to 59 years of age. In contrast to this, the performance of the female officers did not vary significantly across the different age range groups.

When investigating the results by gender there were significant differences in height and body weight between male and female police officers, which is to be expected given previous research investigating gender differences in height and body weight [21]. Given that males tend to have greater skeletal muscle mass [21] and that greater muscle mass is a major factor in gender-related differences in strength [22, 23], the differences in strength (leg/back dynamometer, grip strength), strength endurance (push-ups) and strength influenced movements (e.g. leg power for VJ) observed between the genders in this study are not unexpected. This is reflected in the significant differences between genders in the performance tests measured in this study, including the VJ, leg/back dynamometer, maximum push-ups in 1-min, and grip strength. With the one exception of the 30–39-year-old age group, there were no significant differences between the genders in the sit-up test. This result was not surprising given that some research investigating trunk flexion strength-endurance differences between genders failed to find significant differences when assessing two different trunk flexion exercises [24]. In this study, relatively smaller female sample sizes may have been a notable factor in the differences in findings. Male officers also reported significantly higher MSFT results and a higher predicted VO2 max when compared to the female officers. These results was again expected as males tend to display greater aerobic power and work efficiency when compared to females [25], both of which would positively contribute to MSFT performance.

These gender difference findings are epitomized by fitness requirements of tactical populations that are normalized for gender. For example, in the Australian Army, males are required to complete a higher number of push-up repetitions and complete an aerobic run of 2.4 km in a faster time [26]. Conversely, there are no differences in sit up requirements between genders [26].

When considering the impact of age on performance, it is known that body weight has a tendency to increase with advancing age in the general population [27]. In this study, there appeared to be a similar trend in male officers with body weight increasing with age. However, these increases in body weight with age were only significant in two cases, being the 40–49 and 50–59 year groups being heavier than the 20–29-year group. When comparing mean ages of other research samples in law enforcement, the demographic profiles in this study were similar. For example, in research conducted with male police academy cadets mean ages of 23.7 years [28], 24.6 years [29], and 27.4 years [12] mean weights were reported as being 82.1 kg [28], 82.4 kg [29], and 85.4 kg [12] respectively with the mean male weight in this study for the 20–29 year old age group being 84.85 kg. Likewise, in research including incumbent officers, demographic samples included mean ages of 37.0 year [30], 37.1 year [29], and 37.9 years [31] with a mean mass of 88.7 kg [31], 90.2 kg [30], 94.6 kg [29], respectively compared to a mean weight of 91.4 kg in the 30–39 year age category in this study. Furthermore, research including specialist police (e.g. Special Weapons and Tactics [SWAT] team) mean ages were 33.3 years [32], 34.7 years [33] and 36.5 years [2] with mean body weights of 89.2 kg [32], 91.5 kg [33] and 93.3 kg [2]; again comparative with the findings of this study.

However, it should be noted that in these aforementioned comparative studies, age was not stratified. In the only other known study of LEOs that included body weight by age stratification, no significant differences in weight across the age groups was found (20–29 years = 87.9 ± 12.86 kg: 30–39 years = 91.27 ± 14.56 kg: 40–49 = 93.15 ± 15.26 kg: 50–59 years = 88.26 ± 11.09 kg) with the body weights by age stratification being similar to those of this study [7]. Furthermore, in this study by Dawes et al. [7], there was no significant association between body weight and age (*r* = .046, *p* = .296). One potential reason for the differences in findings between the study by Dawes et al., and this study is the larger standard deviation in body weights reported across all age categories in this study. As such, while body weight may generally increase with age it may not necessarily be by a significant amount in a law enforcement population.

In this study, there was a general decrease in VJ height across age for both genders, although this decrease was only significant in the 20–29-year-old male group. A decrease in VJ height with increasing age is noted in the literature. As an example, in one study Korhonen et al. [34] found that sprint-trained older men performed more poorly in the VJ when compared to younger sprinters (18–33 year group: 52.5 ± 1.62 cm; 40–49 year group: 42.0 ± 0.97 cm; 50–59 year group: 33.1 ± 0.89 cm). A reduction in VJ ability, and hence power, can be linked to the reductions in Type II muscle fiber size, muscle force output, and rate of force development that can occur with aging [34, 35]. Considering this, the results of VJ performance in this study mirror the results associated with body weight and aging whereby the significant difference occurred only with the youngest group (20–29 years). A potential reason for this similarity can be the association between body weight and VJ performance. While percentage of body fat, lean muscle mass and fat mass were not determined in this study, a study by Dawes et al. [36] did find a strong and significant correlation between these composition measures and VJ performance (-.566, .391, and − .369 respectively). Based on these findings, Dawes et al. [36] recommended that increasing lean body mass and decreasing body fat could both positively influence VJ performance. For the female officer population there did appear to be a general decline in VJ performance as age increased, but there were no significant differences. A potential reason for this lack of difference with aging lies in research suggesting that older females may be able to maintain stretch-shortening function better than men [35], a physiological function which could benefit VJ performance.

For the push-up assessment, similar results were found whereby the 30–39, 40–49, and 50–59 year groups for the males all performed significantly fewer repetitions than the 20–29-year group. These results contrast the findings of Dawes et al. [7], who found that 1-min push-up performance did not vary with age in male LEOs. However, this may have occurred because the 30–39, 40–49, and 50–59 year groups from this study performed fewer repetitions than the corresponding age groups in the study by Dawes et al. [7] (30–39 years: 44.65 ± 15.57 repetitions; 40–49 years: 43.92 ± 15.74 repetitions; 50–59 years: 43.71 ± 15.09 repetitions) while the 20–29 year old group performed more repetitions than those in the study by Dawes et al. [7] (44.48 ± 15.47 repetitions). Although not reaching significance, there was a general decline in the female LEOs groups with increasing age. Given this strong trend a lack of significance may have been due to the small sample size of female LEOs across the stratified age groups. This same trend was evident in the sit-up results. For male LEOs there was a trend towards fewer sit-up repetitions across the age group with significant differences only observed between the 20–29-year-old group and all other groups. Likewise, there were no significant differences in sit ups between female officer age groups with a leveling of performance over the age of 30 year.

When considering the aerobic capacity of the male officers, as measured by the MSFT, the 30–39, 40–49, and 50–59 year groups performed significantly poorer than the 20–29-year group. As for several of the other tests, there was a general decline in the MSFT performance and VO2 max for the females, without any significant interactions. On the surface, these results appear to be indicative of the age-related declines that occur in cardiorespiratory function [37]. There could potentially, however, be other limiting factors in the endurance running performance of older LEOs. Anecdotally, many of the older LEOs noted that a limiting factor in the MSFT was not their aerobic fitness, but rather previous injury or joint stress (e.g. knee pain) that caused cessation of the test. Increased body mass and occupations that involve heavy lifting (such as law enforcement work) can lead to joint degeneration and osteoarthritis later in life [38]. This factor could limit the value of using estimated VO2 from this assessment as a measure in this population.

In this study, performance in the isometric strength tests (isometric leg/back pull and grip strength) was similar across all age groups for the male and female officers. As isometric strength can have a tendency to decrease with age [39], it is notable that LEOs appear to be able to maintain this capacity across the different age ranges. Although the requirement for LEOs to have a measure of isometric strength to perform occupation-specific tasks (e.g, including pushing, pulling, dragging, carrying, grappling, defensive tactics, etc [13, 40] may provide a reason for the maintenance of this type of strength, confirmation of these findings and reasons for this isometric strength maintenance require further investigation.

To inform future research as well as provide an interim profile of law enforcement officers, one aim of this research was to provide percentile ranking charts unique to this population. The importance of developing a law enforcement specific profile is clearly identified when the percentile ranking results of this law enforcement population are compared to the percentile rankings of the general population [41]. For example, in the push-up assessment the law enforcement 15^th^ percentile ranking (see Table 5) is equivocal to that of the 50^th^ percentile for the normative populations being: Group 20–29 = 33 repetitions, Group 30–39 = 27 repetitions; Group 40–49 = 21 repetitions and Group 50–59 = 15 repetitions. On this basis, using normative percentile data to guide conditioning and reconditioning of law enforcement may be notably underestimating law enforcement fitness. Consider an injured 35-year-old police officer undergoing reconditioning for return-to-work who can complete 27 push-ups. While this would stand the officer on the 50^th^ percentile of the general population, this officer would only be at a level just below the 20^th^ percentile of his specific population. Conversely, there were very little differences between the general population in sit-up performance with the 50^th^ percentile for the general population generally being in the 45^th^–50^th^ percentile of police officers. For VJ jump results were in-between with the 50^th^ percentile for the general male population ranking from 15^th^ Percentile (40–49 year old and 50–59 year old groups) to the 35^th^ percentile (30–39 year old group) of male police officers.

There were certain limitations for this research that should be noted. Only select physiological characteristics were analyzed in this research: lower-body power, isometric grip of the dominant hand and back/leg strength, upper-body and abdominal strength endurance, and aerobic fitness. It would be of benefit to further analyze the influence of age on other capacities, such as maximal upper- and lower-body strength measured via repetition-maximum tests (e.g. bench press and squat), sprinting speed, and flexibility. This study was cross-sectional in design, and future studies employing a longitudinal approach would be of benefit to better inform impacts of aging on a LEO population. Although typical of LEO and police research [11, 12, 5], there were a limited number of female officers in this sample and as such additional research studies or larger cohorts are needed to support the finding presented in this study.

## Conclusion

The findings of this study suggest that, on average, male LEOs tended to be heavier, taller and displayed greater lower limb power, dominant hand grip strength, upper limb muscular endurance and metabolic fitness than female officers with no consistent differences in trunk muscular endurance. Furthermore, across both genders it appears that certain physical characteristics may decline with age in LEOs. This included lower-body power, upper-body strength endurance, and aerobic capacity as measured by the MSFT. Based on these findings, and within the context of the limitations, an initial profile of male and female LEOs across different age ranges: 20–29, 30–39, 40–49, 50–59, and 60–69 years of age was established.

Percentile rankings, which were found to have elements very specific to this population when compared to the general population, were provided based on the data, including VJ, hand grip strength, number of push-ups and sit-ups completed in 1-min; leg/back chain dynamometer; and number of MSFT shuttles. These population specific percentile rankings can be used to inform expectations of performance above those generated for the general population in an LEO population.

## Abbreviations

ANOVA: Analysis of Variance
cm: Centimetres
in: Inches
kg: Kilograms
lbs: Pounds
LEO: Law Enforcement Officer
MSFT: Multi-Stage Fitness Test
SPSS: Statistical Package for the Social Sciences
VJ: Vertical Jump
VO2: Volume of Oxygen consumed
yrs: Years
